# Effect of vitamin D deficiency on the development of postoperative atrial fibrillation in coronary artery bypass patients

**DOI:** 10.15171/jcvtr.2016.29

**Published:** 2016-12-27

**Authors:** Safa Gode, Timuçin Aksu, Aylin Demirel, Murat Sunbul, Mehmet Gul, Ihsan Bakır, Mehmet Yeniterzi

**Affiliations:** ^1^Department of Cardiovascular Surgery, Istanbul Mehmet Akif Ersoy Thoracic and Cardiovascular Surgery Training and Research Hospital, Istanbul, Turkey; ^2^Department of Cardiology, Marmara University School of Medicine, Istanbul, Turkey; ^3^Department of Cardiology, İstanbul Mehmet Akif Ersoy Thoracic and Cardiovascular Surgery Training and Research Hospital, Istanbul, Turkey

**Keywords:** Vitamin D, Atrial Fibrillation, Coronary Artery Bypass Graft

## Abstract

***Introduction:*** Various factors may be responsible for the development of postoperative atrial fibrillation (POAF) in coronary artery bypass graft (CABG) patients. In our study, we demonstrated the effect of vitamin D deficiency on the development of POAF.

***Methods:*** In this prospective case control study, patients undergoing elective, isolated CABG were considered. A total of 15 patients (16.6%) who developed POAF during the first five days after surgery made up the POAF group. Seventy-five patients that had a sinus rhythm in the same period were the non-POAF group. The two groups were compared statistically in terms of laboratory, clinical, echocardiographic, operative, and postoperative parameters.

***Results:*** All patients were in sinus rhythm at discharge. The baseline characteristics of the study groups were comparable. The POAF group had a lower vitamin D level than the non-POAF group (9.0 ± 5.0 and 15.0 ± 8.4 ng/mL, respectively; *P*=0.007). In the POAF group, the patients’ left atrium diameter and incidence of hypertension (HT) were higher than those of the non-POAF group.

***Conclusion:*** Incidence of POAF was significantly higher in patients with vitamin D deficiency or insufficiency than the patients with vitamin D level in normal range. Therefore vitamin D deficiency or insufficiency may be a predictor of POAF in patients with CABG.

## Introduction

Postoperative atrial fibrillation (POAF) is a common complication after coronary artery bypass graft (CABG). It affects about 30%–60% of patients who undergo CABG.^1,2^ POAF has also been associated with both short and long term complications.^2,3^ This arrhythmia is observed most frequently in the first five days of the postoperative period, especially during the first 24–72 hours.^4^ POAF increases the risk of cerebrovascular accident, which extends the duration of intensive care unit and hospital stays.^5^ This complication also increases the cost of surgical treatment. Some of the risk factors associated with the development of POAF are advanced age, right atrial manipulation, atrial myocardial ischemia, cardiopulmonary bypass (CPB), and prolonged aortic cross-clamping time.^6,7^

The incidence of vitamin D deficiency is high in Western populations and has been considered a global health problem, particularly in the elderly.^8,9^ Vitamin D is associated with bone formation and calcium metabolism in the cardiovascular system. Additionally, inadequate vitamin D levels lead to an increased risk of developing cardiovascular disease, hypertension (HT), and obesity.^10,11^ Vitamin D influences human health via several different mechanisms. In brief, vitamin D plays a role in the inhibition of the renin-angiotensin-aldosterone system (RAAS) and has also been associated with inflammatory processes. Both are involved in the pathophysiology of atrial fibrillation (AF). Therefore, vitamin D can influence the etiology of POAF.^12^

The aim of this prospective study was to investigate the effect of preoperative vitamin D deficiency and insufficiency to the development of POAF that was defined as an episode of AF recorded on continuous telemetry and verified using 12-lead electrocardiogram within the first five days postoperatively in patients with on pump CABG.

## Patients and Methods

**Figure 1:**
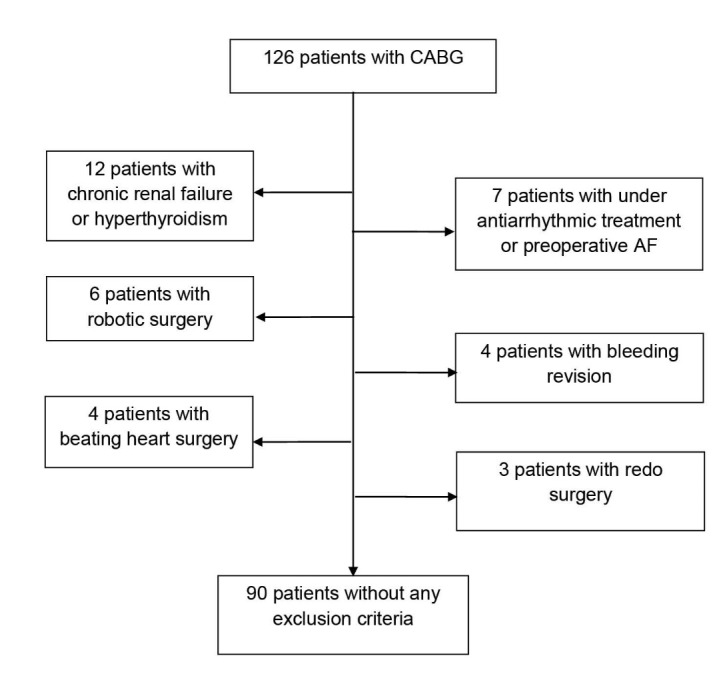
Patients flow chart. AF: atrial fibrillation, CABG:coronary artery bypass graft.

In this prospective, case control study, patients undergoing elective primary isolated CABG at the Department of Cardiothoracic Surgery of Istanbul Mehmet Akif Ersoy Thoracic and Cardiovascular Surgery Training and Research hospital between January 2016 and March 2016 were considered. Exclusion criteria included preoperative AF, current using vitamin D supplement and antiarrhythmic treatment (amiodarone, etc.), beating heart surgery, robotic surgery, redo surgery, bleeding revision, chronic renal failure, and hyperthyroidism. Thirty-six of the 126 patients were excluded from the study due to these criteria (Figure 1). Therefore, 90 final patients were enrolled in the study.

Chronic renal failure was defined as a blood creatinine level of >1.5 mg/dL or if the patient were currently enrolled in a hemodialysis program. Hyperthyroidism was defined as a blood thyroid stimulating hormone (TSH) level of <0.4 mg/dL or if the patient were on antithyroid medication. All patients were operated on by the same surgery team under CPB in standard fashion using mild hypothermia (32–34°C) in this study.

The study population included 65 males and 20 females, with a mean age of 58.1 years. Patients were monitored continuously by five-lead telemetry 24 hours in a day from surgery until the fifth day postoperatively. POAF was defined as an episode of AF recorded postoperatively on continuous telemetry, and AF was verified using 12-lead electrocardiogram (EKG). AF diagnostic criteria including that absence of discrete P waves, the RR intervals follow unrepetitive form; they have been labeled as “irregularly irregular” and the ranges of ventricular rate usually between 90 and 170 beats/min were considered for POAF by a cardiologist in our study. The participants were divided into two groups. Patients who experienced AF at any time during the first five days after the operation were placed in the POAF group (n = 15), while patients with a sinus rhythm in the same period were considered the non-POAF group (n = 75).

This study was approved by the ethics committee of our hospital. Additionally, we also obtained the patients’ written informed consent to be included in the study. The two groups were compared in terms of their clinical diagnoses and usage of the following medications upon admission: angiotensin converting enzyme inhibitors, beta blockers, calcium channel blockers, and statins (Table 1). A comparison was also performed between the groups in terms of their parathyroid hormone (PTH), vitamin D and other laboratory and echocardiographic parameters (Table 2), as well as their operative and postoperative parameters (Table 3).

**Table 1:** Comparison of the groups in terms of clinical and admission medications parameters

	**Group with POAF (n=15)**	**Group with non-POAF (n=75)**	***P***
Age (y), ( ± SD)	59.1 ± 5.4	58.4 ± 9.1	0.659
Gender (female), n (%)	5 (33)	15 (33)	0.313
Smoking (pocket/year) ( ± SD)	19.0 ± 22.1	21.0 ± 20.7	0.740
Alcohol consumption (n, %)	0 (0)	3 (4)	0.575
BSA ( ± SD)	1.9 ± 0.1	1.9 ± 0.2	0.567
DM, n (%)	10 (66)	35 (46.6)	0.157
HT, n (%)	11 (73.3)	32 (42.6)	0.029^a^
COPD, n (%)	0 (0)	13 (17.3)	0.115
ACEi use, n (%)	7 (46.6)	35 (46.6)	0.613
Beta blocker use, n (%)	10 (66.6)	55 (73.3)	0.753
Calcium channel blocker, n (%)	7 (46.6)	24 (32)	0.373
Statin use, n (%)	13 (86.6)	57 (76)	0.506

Abbreviations: ACEi, angiotensin converting enzym; BSA, body surface area; CCB, calcium channel bloker; COPD, chronic obstructive pulmonary disease; DM, diabetes mellitus; HT, hypertension; POAF, postoperative atrial fibrilation; SD, standard deviation. ^a^ Statistically significant.

**Table 2:** Comparison of the groups in terms of laboratory and echocardiography parameters

	**Group with POAF (n=15)** **mean ± SD**	**Group with non-POAF (n=75)** **mean ± SD**	***P***
WBC (k/mm^3^)	8.6 ± 1.5	8.5 ± 2.1	0.946
Hemoglobin (g/dL)	13.7 ± 1.8	14.8 ± 3.8	0.298
CRP (mg/dL)	7.8 ± 8.2	6.3 ± 6.8	0.610
Ca (mg/dL)	9.6 ± 0.4	10.0 ± 3.1	0.642
PTH (pg/mL)	75.9 ± 86.2	51.7 ± 41.9	0.304
25(OH) vitamin D (ng/mL)	9.0 ± 5.0	15.0 ± 8.4	0.007^a^
Creatinin (mg/dL)	1.3 ± 1.2	1.0 ± 0.5	0.244
TSH (mcIU/mL)	2.2 ± 1.3	2.2 ± 1.9	0.871
LDL (mg/dL)	97.6 ± 41.4	117.4 ± 41.7	0.106
HDL (mg/dL)	44.5 ± 6.9	44.8 ± 12.1	0.912
Total cholesterol (mg/dL)	173.5 ± 42.3	192.1 ± 48.1	0.168
Triglycerides (mg/dL)	137.6 ± 58.8	153.6 ± 65.1	0.380
EF (%)	52.7 ± 10.3	54.7 ± 9.3	0.452
LV end diastolic diameter (mm)	32.2 ± 6.7	32.6 ± 7.1	0.832
LV end sistolic diameter (mm)	47.9 ± 6.6	45.6 ± 7.7	0.282
Septum (mm)	11.8 ± 2.1	10.8 ± 1.6	0.100
PW (mm)	10.9 ± 1.6	10.4 ± 3.1	0.554
RAD (mm)	37.1 ± 4.5	37.1 ± 5.0	0.992
LAD (mm)	38.1 ± 3.6	36.0 ± 4.6	0.034^a^

Abbreviations: CRP: C reactive protein, EF: ejection fraction, HDL: high-density lipoprotein, LAD: left atrial diameter, LDL: low-density lipoprotein, LV: left ventricle, POAF: postoperative atrial fibrilation PTH: parathyroid hormone, PW: posterior wall, SD: standard deviation, TSH: thvroid-stimulating hormone, RAD: right atrial diameter, WBC: White blood cell. ^a^ Statistically significant.

**Table 3:** Comparison of the groups in terms of operative and postoperative parameters

	** Group with POAF (n=15) ** ** mean ± SD **	** Group with non-POAF (n=75) ** ** mean ± SD **	***P***
Bypassed vessel count (n)	3.1 ± 0.7	2.8 ± 1.0	0.290
TPT (min)	83.6 ± 28.0	82.9 ± 25.0	0.927
ACCT (min)	44.1 ± 19.1	42.1 ± 15.4	0.663
Time of Intubation (h)	10.7 ± 3.8	8.7 ± 3.2	0.083
Time of ICU (days)	1.1 ± 0.4	1.2 ± 0.5	0.602
Blood loss per patient (mL)	973.3 ± 543.8	756.7 ± 529.2	0.153

Abbreviations: ACCT: aortic cross clamp time, ICU: intensive care unit, SD: standart deviation, TPT: total perfusion time.

### Measurements method of vitamin D and biochemical parameters

Blood samples were drawn from the antecubital vein in the morning before 10:00 am after overnight fasting. Blood was drawn into standardized tubes containing dipotassium ethylenediaminetetraacetic acid (EDTA) to be stored at room temperature. The levels of vitamin D and other biochemical parameters were analysed in biochemistry laboratory within 90 min of venipuncture.

### Statistical analysis

Statistical examination were applied using SPSS 16.0 statistical package for Windows. Sustained data were expressed as mean ± standard deviation (SD) while categorical data were presented as percentage. Chi-square test was used for comparison of categorical variables while *t* test or Mann-Whitney U test were used to compare parametric and nonparametric constant variables, respectively. Normal distribution was assessed by Kolmogorov-Smirnov test. Logistic regression analysis was performed to determine the independent predictors of postoperative AF. A value of *P* < 0.05 was considered statistically significant.

Effect size was defined according to Post-hoc power analysis.^13,14^ We calculated effect sizes of vitamin d deficiency and insufficiency. Effect sizes of vitamin D deficiency and insufficiency were 77.6% and 44.9% respectively. Our results demonstrated that those parameters had large or very large effect size.

## Results

Of the 90 included patients, 15 (16.6%) developed POAF during the first five days postoperatively. Therefore, there were 15 patients in the POAF group and 75 patients in the non-POAF group. The mean ages were 59.1 ± 5.4 and 58.4 ± 9.1 in the non-POAF group and in the POAF group, respectively. A comparison was made between groups based upon their clinical diagnoses and admission medications, including ACE inhibitors, beta blockers, calcium channel blockers and statins, and the incidence of HT was significantly higher in the POAF group (*P* = 0.029) (Table 1). In terms of laboratory parameters, the vitamin D level was significantly higher in patients without POAF than in those with POAF (*P* = 0.007). The other laboratory parameters were statistically similar in the two groups (Table 2). The group with POAF showed a significantly higher left atrial diameter (LAD) than the group without POAF (*P* = 0.034). There was no significant difference in terms of the ejection fraction (EF) or any other echocardiographic parameters between the two groups (Table 2). Additionally, no difference was reported in the number of bypassed vessels, total perfusion time (TPT), and aortic cross clamp time (ACCT) (Table 3). Among the postoperative risk factors, the duration of intubation, blood loss per patient, and the length of intensive care unit (ICU) and hospital stays were statistically the same in the two groups (Table 3).

Also all patients were evaluated in terms of level of vitamin D. Number of patients with normal vitamin D level (above 30 ng/mL) were 9 and 0 in non POAF group and POAF group respectively. There were 44 and 5 patients with insufficient of vitamin D (between 10-30 ng/mL) and 22 and 10 patients with deficient of vitamin D (less than 10 ng/mL) in non POAF group and POAF group respectively. When two groups were compared in terms of level of vitamin D, deficiensy and insuficiensy of vitamin D were statistically high in POAF group than non POAF group (*P* = 0.017; Table 4).

**Table 4:** The distribution of patients according to the presence of new-onset AF and vitamin D status

**Level of 25(OH) vitamin D**	** Group with POAF ** ** n (%) **	** Group with non-POAF ** ** n (%) **	***P***
Deficiency (<10 ng/mL)	10 (66,7)	22 (29,3)	P=0.017^a^
Insufficiency (10-30 ng/mL)	5 (33,3)	44 (58,7)	
Normal (>30 ng/mL)	0	9 (12)	

Abbreviations: POAF: postoperative atrial fibrilation; AF, atrial fibrillation. ^a^ Statistically significant.

Table 5 shows the results of univariate analysis of factors related to the development of POAF. The unadjusted univariate analysis demonstrated that the risk factors for AF were HT (*P* = 0.038) and vitamin D deficiency (*P* = 0.014) (odds ratio [OR]: 3.695, 95% CI: 1.077–12.674, *P* = 0.038; OR: 0.858, 95% CI: 0.759–0.970, *P* = 0.014, respectively). Other variables were not significantly associated with the development of POAF. After eliminating variables that were closely related to others, these independent risk factors for POAF were adopted as confounders in the logistic regression model for the multivariate analysis.

**Table 5:** Univariate logistic regression analysis to determine the predictors of POAF

	Odds ratio	95% CI	*P*
Age	1.011	0.947-1.080	0.747
Gender	0.508	0.151-1.712	0.275
HT	3.695	1.077-12.674	0.038^a^
DM	2.286	0.713-7.331	0.164
Total cholesterol	0.991	0.979-1.004	0.170
Creatinine	1.915	0.906-4.047	0.089
EF	0.978	0.924-1.036	0.448
LAD	1.016	0.999-1.033	0.072
25(OH) vitamin D	0.858	0.759-0.970	0.014^a^

Abbreviations: DM, diabetes mellitus; EF, ejection fraction; HT, hypertension; LAD, left atrial diameter. ^a^ Statistically significant.

**Figure 2:**
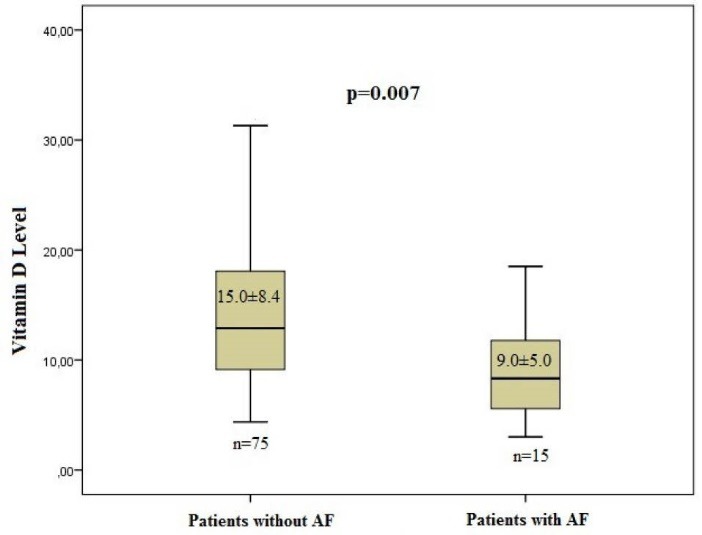
Comparison of the groups in terms of vitamin D level.

According to Vittinghoff et al^15^ the variables those *P* < 0.10 or *P* < 0.15 in univariate analyses may be statistically significant in multivariate analyses. Therefore these variables are included to multivariate analyses. For this reason we included the LAD, creatinine, cholesterol and DM to the multivariate analyses. Only one factor was identified as an independent predictor of postoperative AF after CABG surgery in multivariate analysis: the level of vitamin D (OR: 0.856, 95% CI: 0.751–0.976, *P* = 0.020) (Table 6; Figure 2).

**Table 6:** Multivariate logistic regression analysis to determine the independent predictors of POAF

	**Odds Ratio**	**95% CI**	***P***
HT	3.738	0.811-17.224	0.091
DM	1.379	0.328-5.806	0.661
Total Cholesterol	0.991	0.977-1.006	0.227
Creatinine	1.958	0.681-5.632	0.213
LAD	1.077	0.925-1.254	0.338
25(OH) vitamin D	0.856	0.751-0.976	0.020a

Abbreviations: DM, diabetes mellitus; EF, ejection fraction; HT, hypertension; LAD, left atrial diameter. ^a^ Statistically significant.

### Clinical outcomes

The patients who developed POAF were treated medically with intravenous amiodarone initially and then the tablet form thereafter. Electrical cardioversion was not required in any patients with POAF. Duration of POAF was so variable from 139 minutes to 16 hours but there were not second episode of AF in patients and all patients were in sinus rhythm at discharge. There was no mortality during the early postoperative period. One stroke and one transient ischemic attack occurred postoperatively in POAF group. Acute renal insufficiency developed in two patients whose renal functions had been normal preoperatively in non POAF group, but they did not require hemodialysis. Sternal wound infection was observed in one patient in each group, and one of them underwent sternal revision in POAF group. There was no pericardial effusion requiring intervention in any group.

## Discussion

This study demonstrated the relationship between vitamin D deficiency and POAF development. The patients who developed POAF had a significantly lower mean concentration of serum 25 (OH) vitamin D than the patients with sinus rhythm (Figure 2).

When cardiac myocytes were induced, positively charged ions (Na^+^) enter to cardiomyocyte and action potential begins. Thereafter inward calcium currents provide to the plateau phase of membrane potential which determine the duration of action potential. Myocytes are not sensitive to electrical impulse in the plateau phase. Extensively increased intracellular calcium may be toxic. Therefore, the adaptive mechanisms that down regulate the calcium channels play an important role in decreasing the intracellular calcium. Thereupon, the duration of action potential is reduced leading to early reactivation and triggering AF.

POAF is one of the most common causes of morbidity following cardiac surgery.^16,17^ Although AF is a frequent postoperative complication, the incidence of POAF in patients undergoing cardiac surgery is still unknown. As reported in the literature, AF occurs most often in the first week postoperatively, and the incidence range is between 30%–60%.^18,19^ The frequency of POAF was 16.6% in our study. This result was relatively small compared with the existing literature and may have been related to the strict elimination criteria used in our study population.

Unlike non-surgical cases, AF that occurs in the postoperative period does not have a well-defined cause. Various risk factors are related to the increased incidence of AF in patients postoperatively, including paroxysmal AF history, previous myocardial infarction, DM, HT, chronic obstructive pulmonary disease (COPD), advanced age, discontinuation of beta-adrenergic drugs before surgery, ACCT, postoperative ischemia and vasoactive amines.^20,21^

The other predisposing factor for the development of AF is vitamin D deficiency.^22^ Vitamin D own a potential connection with cardiac diseases. There are so much vitamin D receptors in most cardiac and vascular tissues, including cardiomyocytes, vascular smooth muscle, and the endothelium. New studies have demonstrated that vitamin D inhibits the RAAS, suppresses cardiac myocyte hypertrophy, and decreases inflammation.^23,24^ Whole these mechanisms may conduce to the increased cardiovascular risk including AF associated with vitamin D deficiency.

Previous studies have described the role of vitamin D deficiency in the onset of AF by various mechanisms. Canning et al^24^ reported that vitamin D regulates inflammatory responses and up-regulates the expression of anti-inflammatory cytokines as IL-10 in their in vitro experiments. In addition, an activated RAAS can lead to AF due to oxidative stress and inflammation.^25^ Vitamin D prevents AF by inhibiting RAAS activity.^26^ The other hypothesis for how AF develops in vitamin D deficiency is that tissue angiotensin II can contribute to changes in the atrial structure by inducing apoptosis of cardiomyocytes.^27^ Consequently angiotensin is inhibited and atrial structure is protected by vitamin D.

Thompson et al^28^ demonstrated that vitamin D prolongation to duration of action potential by directly and atrial specific antiarrhythmic effect. Confirmingly, Hanafy et al^29^ performed an electrophysiological study with rabbits related to vitamin D. They observed that 1,25[OH]_2_D has direct electrophysiological and mechanical effects on the atrium, which can prevent or terminate AF by prolongation of the action potential duration. Further, Demir et al^30^ observed that vitamin D deficiency reduced the vulnerability to AF by causing an increase in transforming growth factor B1 expression, improving atrial fibrosis, and conduction heterogeneity. And they emphasized that vitamin D deficiency were more related to non valvular AF than valvular AF.

It is known that PTH causes an increase in intracellular calcium levels by reducing calcium intake to the cardiomyocytes and decreasing reuptake to the sarcoplasmic reticulum.^31^ Chen et al^22^ determined that PTH level was higher in the AF group than in the non-AF group. Therefore high PTH level due to vitamin D deficiency leads to AF resulting in intracellular calcium overload.

Calcium is an electrolyte that effective in trigger of AF and in electrophysiological remodeling. High level of calcium in the atrial myocytes plays a role in the formation and progression of the AF owing to abbreviating action potential time and atrial refractory period.^32^ Also calcium provides to development of atrial enlargement and electrophysiological remodeling.^33^ Contrary to this, there were no difference statistically between the POAF group and non POAF group in terms of calcium and PTH in our study and the both parameters were normal range.

HT was also a predisposing factor in the development of POAF in our study. However, the effect of HT on POAF could not be determined using logistic regression analyses. According to a study of Tinica et al^34^ there was no difference between the AF group and the non-AF group in terms of preoperative HT in cardiac surgery patients. Additionally, these same authors demonstrated that the EF was significantly lower in patients with POAF and LAD was the same statistically in their two groups. Contrary to this finding, we observed that LAD was significantly higher in patients with POAF, but this difference disappeared in the multivariate analyses.

In this study, we demonstrated that deficiency or insufficiency of vitamin D was related to POAF. If there is a lack of this vitamin preoperatively, it must be replaced with 5–15 μg/d to prevent POAF in patients with CABG.

## Limitations

Our first study limitation was the relatively small sample size. While our study was designed prospectively, we only had EKG data on patients for the first five postoperative days. A longer follow-up period with patients would allow us to obtain a better picture of the effects of vitamin D on AF in patients with CABG. In addition, further prospective, randomized, double-blind, and large-scale clinical trials will be necessary to confirm our results.

## Conclusion

The level of vitamin D may be important in terms of development of POAF in CABG patients. POAF was more common in patients with vitamin D deficiency or insufficiency than the patients with enough vitamin D level in this study. Therefore it may be a predictor of POAF in patients with CABG.

## Competing interests

Authors declare no conflict of interest in this study.

## Ethical approval

Written informed consent was obtained from each subject and the institutional ethics committee approved the study protocol.
